# Risk-Adapted, Individualized Treatment Strategies of Myelodysplastic Syndromes (MDS) and Chronic Myelomonocytic Leukemia (CMML)

**DOI:** 10.3390/cancers13071610

**Published:** 2021-03-31

**Authors:** Jan Philipp Bewersdorf, Amer M. Zeidan

**Affiliations:** Department of Internal Medicine, Section of Hematology, Yale University School of Medicine, 333 Cedar Street, P.O. Box 208028, New Haven, CT 06520-8028, USA; jan.bewersdorf@yale.edu

**Keywords:** Myelodysplastic syndrome, MDS, chronic myelomonocytic leukemia, CMML, genetics, management

## Abstract

**Simple Summary:**

Myelodysplastic syndrome (MDS) and chronic myelomonocytic leukemia (CMML) are two blood cancers with variable symptoms of low blood counts (fatigue, bleeding, infection risk) and risk of progression to acute myeloid leukemia. Management decisions should be guided by individual patient and disease characteristics and based on validated risk stratification tools. Supportive care with red blood cell transfusions and medications to stimulate blood cell production remains the mainstay of therapy for lower-risk MDS and CMML patients. For higher-risk patients, a bone marrow transplant is the only potentially curative option, but most patients are not candidates for this intensive therapy. In this case, the hypomethylating agents (HMA) azacitidine and decitabine are standard of care. However, response rates to HMA are low and responses are only transient highlighting the need for novel approaches. While an oral version of decitabine has been recently approved, several targeted therapies are in development, but none has been approved to date.

**Abstract:**

Myelodysplastic syndrome (MDS) and chronic myelomonocytic leukemia (CMML) are two distinct blood cancers with a variable clinical symptom burden and risk of progression to acute myeloid leukemia. Management decisions should be guided by individual patient and disease characteristics and based on validated risk stratification tools. While supportive care with red blood cell transfusions, erythropoiesis-stimulating agents, and iron chelation remains the mainstay of therapy for lower-risk (LR)-MDS patients, luspatercept has recently been approved for transfusion-dependent anemic LR-MDS patients ending a decade without any new drug approvals for MDS. For higher-risk patients, allogeneic hematopoietic cell transplant (allo-HCT) remains the only curative therapy for both MDS and CMML but most patients are not eligible for allo-HCT. For those patients, the hypomethylating agents (HMA) azacitidine and decitabine remain standard of care with azacitidine being the only agent that has shown an overall survival benefit in randomized trials. Although early results from novel molecularly driven agents such as IDH1/2 inhibitors, venetoclax, magrolimab, and APR-246 for MDS as well as tagraxofusp, tipifarnib, and lenzilumab for CMML appear encouraging, confirmatory randomized trials must be completed to fully assess their safety and efficacy prior to routine clinical use. Herein, we review the current management of MDS and CMML and conclude with a critical appraisal of novel therapies and general trends in this field.

## 1. Introduction

Myelodysplastic syndromes (MDS) are a heterogeneous group of myeloid malignancies that are characterized by dysplasia of myeloid elements in the bone marrow, ineffective hematopoiesis leading to cytopenias, and a variable risk of progression to acute myeloid leukemia (AML) [1,2]. As clinical manifestations and prognosis are variable, several risk stratification tools have been developed to tailor management decisions to the individual patient with the International Prognostic Scoring System (IPSS) and its revised version IPSS-R being the most commonly used scoring tools [3,4,5]. Recently, those clinical-pathologic scoring systems have been supplemented by genetic and molecular assessments that improve risk stratification but may also be predictive of response to specific therapies such as *SF3B1* mutations as a biomarker of response to luspatercept [6,7,8].

Overlap syndromes between MDS and myeloproliferative neoplasms (MPN) are rare and encompass various disease subtypes as defined by the 2016 World Health Organization classification of myeloid neoplasms and acute leukemia [9]. These include chronic myelomonocytic leukemia (CMML), atypical chronic myeloid leukemia, juvenile myelomonocytic leukemia, MDS/MPN with ring sideroblasts, and thrombocytosis (MDS/MPN-RS-T), and MDS/MPN unclassifiable with CMML being the most common subtype of MDS/MPN overlap syndromes [9]. Recently, the genetic landscape of CMML has been increasingly elucidated and mutations in *TET2* (~60%), *SRSF2* (~50%), *ASXL1* (~40%), and *SETBP1* (~15%) are common but not specific for CMML [10,11].

Treatment decisions for both MDS and CMML should focus on the individual patient and options range from observation to supportive care with red blood cell (RBC) transfusions and erythropoiesis-stimulating agents (ESA) to hypomethylating agents (HMA) and ultimately allogeneic hematopoietic cell transplant (allo-HCT) [1,10,12,13,14].

## 2. Risk Stratification in MDS and CMML as the Basis for Treatment Selection

Treatment selection for the individual MDS patient is driven by disease risk and symptom burden. Both in routine clinical practice and for clinical trial enrollment IPSS and IPSS-R are the most commonly used risk stratification tools, which predict both median overall survival (OS) and 25% AML progression rate [4,5]. However, both scores are only validated for the time of diagnosis and have limitations in specific subgroups of MDS patients such as those with therapy-related or lower-risk MDS (LR-MDS) or at the time of HMA failure for which specific scoring systems have been developed but are not widely used [15,16,17].

More recently, molecular testing has become more widely available and somatic mutations in genes such as *EZH2, SF3B1,* and *TP53* have been shown to provide additional prognostic information when added to conventional clinical-pathologic scores [18,19]. As exemplified by *TP53,* the prognostic impact of mutations should not be interpreted in isolation as the prognostic impact of *TP53* mutations, for example, depends on the presence of a complex karyotype or the specific type of *TP53* mutation [6,20]. With the exception of *SF3B1* mutations, the influence of somatic mutations on the response to HMA treatment is controversial with some studies having identified *TET2* mutations as predictive markers for response to HMA [21,22,23]. 

**Figure 1:**
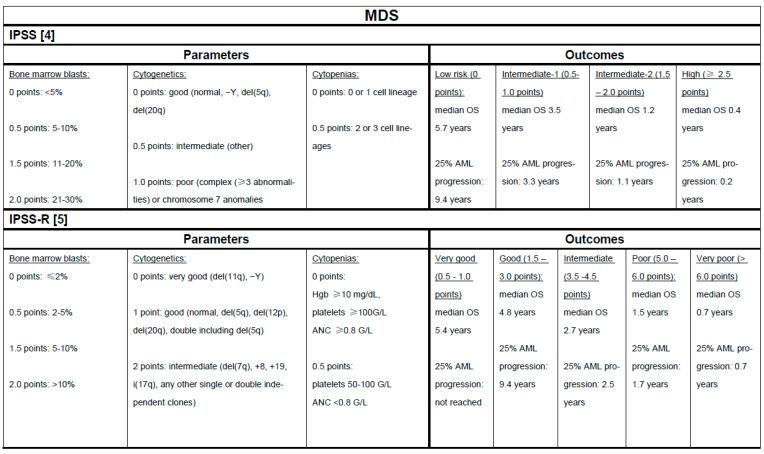

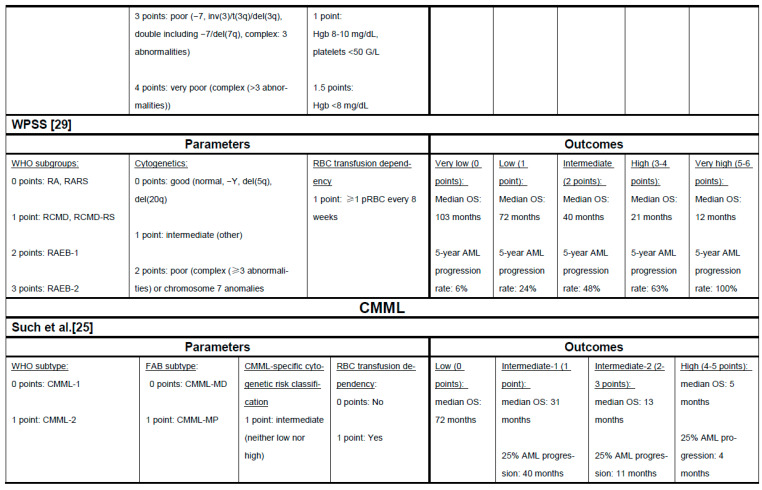

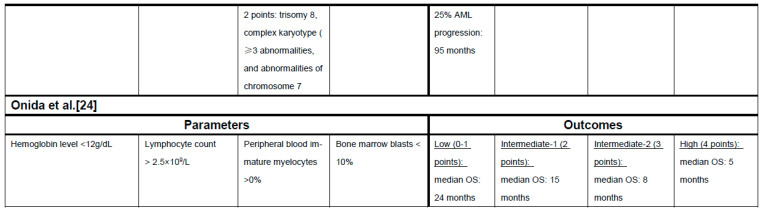
Selected clinical-pathological risk stratification tools for Myelodysplastic syndrome (MDS) and chronic myelomonocytic leukemia (CMML) [4,5,24,25,29]. Underlines: indicate the subheading in each column.

**Figure 2:**
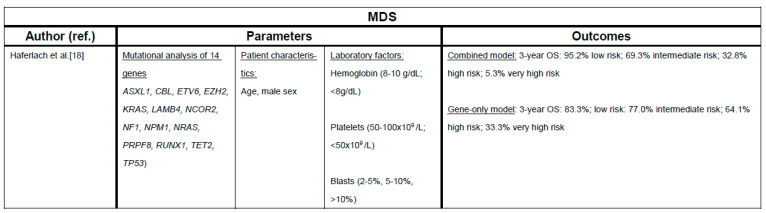

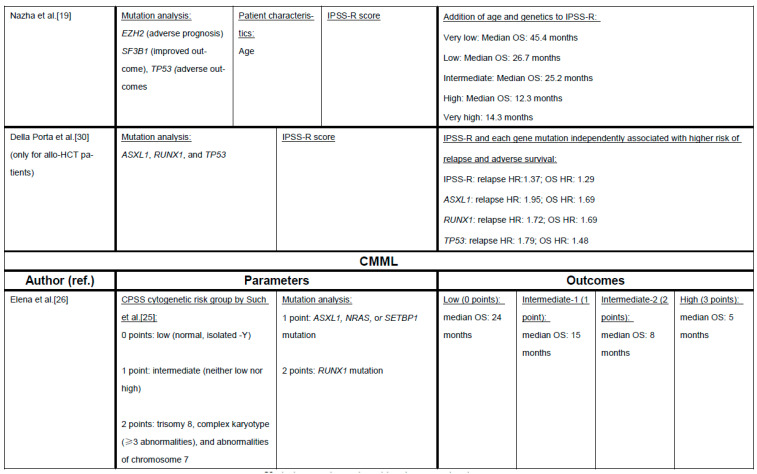
Selected molecular risk stratification tools for MDS and CMML. Underlines: indicate the subheading in each column [18,19,26,30].

Conventional risk stratification tools such as IPSS and IPSS-R are of limited use for CMML patients and dedicated scores such as the MD Anderson prognostic system (MDAPS) and the CMML-specific prognostic scoring system (CPSS) have been developed [24,25]. Following advances in molecular diagnostics, additional prognostic scoring systems incorporating molecular data have been developed with mutations in *RUNX1, NRAS, SETBP1,* and *ASXL1* having been associated with adverse outcomes [26,27,28]. Figure 1 and Figure 2 provide a summary of selected risk stratification tools.

## 3. Treatment Algorithm for MDS

**Figure 3:**
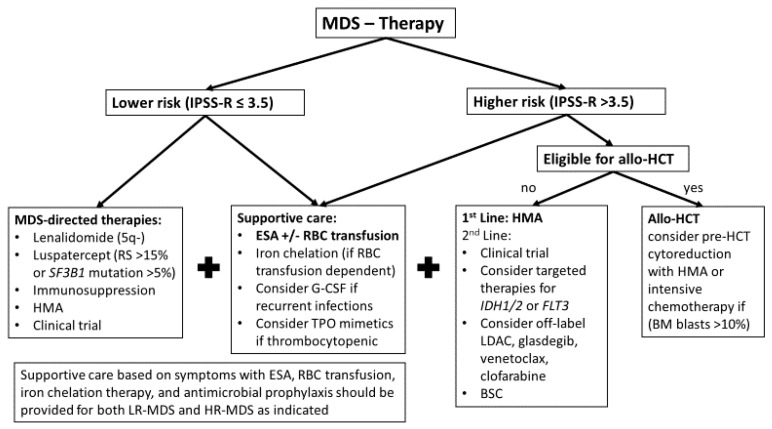
Potential treatment algorithm for MDS. Treatment selection for MDS patients depends on individualized risk assessment using validated scoring systems such as IPSS-R. All patients with MDS should receive supportive care based on their symptoms with erythropoiesis-stimulating agents (ESA), blood product transfusion (red blood cells [RBC] and platelets), iron chelation therapy, and antimicrobial prophylaxis if neutropenic [1,33]. TPO mimetics for thrombocytopenic patients and G-CSF in patients with recurrent infections can be considered as supportive care for MDS patients as well. However, the use of the latter two should be carefully considered due to concern for accelerated AML transformation with growth factor use. For patients with lower-risk MDS, especially if they are refractory to ESA and RBC-transfusion-dependent lenalidomide, luspatercept, immunosuppressive therapy, hypomethylating agents (HMA), or enrollment in clinical trials are potential options based on patient and disease characteristics [2,31]. Allogeneic hematopoietic cell transplant (allo-HCT) remains the only potentially curative therapeutic modality for MDS and all patients with higher-risk MDS (and selected lower-risk patients) should be considered for this curative modality [2]. If patients are allo-HCT eligible, pre-transplant cytoreduction with HMA or intensive chemotherapy can be considered if bone marrow blast percentage is >10%. For non-transplant patients, HMAs remain the standard of care [2,31]. In patients with HMA-failure, clinical trials, as well as the best supportive care (BSC) only are 2nd line modalities [2]. Data are limited on targeted therapies with IDH1/2 or FLT3 inhibitors. In the absence of the clinical trials option, the off-label use of low-dose cytarabine (LDAC), glasdegib, venetoclax, or clofarabine could be considered as a last line of therapy.

Treatment of patients with LR-MDS as defined by IPSS-R score of ≤3.5 points are treated along a spectrum reaching from surveillance to supportive care with ESA and blood transfusions as well as HMA, immunosuppressive therapy, or lenalidomide based on symptom burden and disease characteristics [2,31,32,33]. Figure 3 provides a potential treatment algorithm for MDS patients adapted from European and American panel recommendations and expert opinions [1,2,31,33,34]. 

### 3.1. Lower-Risk MDS

Anemia is the most common symptom in patients with LR-MDS and is treated symptomatically based on individual patient factors [1,35]. Supportive care with ESA is the standard of care for patients with serum erythropoietin (EPO) levels below 200 U/L with studies showing a decreasing efficacy with higher serum EPO levels [33,36,37]. Other predictors of a higher likelihood of response to ESA include lower IPSS scores, shorter disease duration, and a lower bone marrow blast percentage [36,37,38]. While ESA have been shown to improve quality of life, and treatment with the combination of ESA and the granulocyte colony-stimulating factor (G-CSF) can be more effective than with ESA alone, overall response rates (ORR) for ESA +/− G-CSF have been reported to be only 34–46% in clinical trials and prospective studies [39,40,41]. Additionally, responses to ESA are only transient with median response durations of 11–23 months [39,40]. As such, many patients will eventually become RBC transfusion-dependent and additional supportive care measures such as iron chelation are necessary. In the recently published randomized TELESTO trial, iron chelation with deferasirox has been shown to prolong event-free survival (EFS; defined as a composite of worsening cardiac function, hospitalization for congestive heart failure, liver function impairment, cirrhosis, and transformation to AML) compared to placebo in transfusion-dependent patients with low- or intermediate-1 risk MDS (3.9 years [95% confidence interval [CI]: 3.2–4.3 years] vs. 3.0 years [2.2–3.7 years]; hazard ratio [HR]: 0.64 [0.42–0.96]) [42]. Of note, this difference was primarily driven by a lower rate of heart failure hospitalizations, and no OS benefit was shown [42]. Additionally, the study was limited by slow accrual which necessitated conversion from a phase III to a phase II design and enrollment of only 210 instead of the planned 630 patients [42].

In the 5–15% of MDS patients with del(5q), lenalidomide has been shown in clinical trials to lead to transfusion independence in 67% of patients with 45% achieving a complete cytogenetic response in a non-randomized trial [43]. However, neutropenia and thrombocytopenia can have dose—and treatment-limiting side effects which have been reported in up to 55% and 44% of patients, respectively [43]. A statistically significant improvement in transfusion independence (56.1% and 42.6% vs. 5.9%; both *p* < 0.001) and complete cytogenetic response rates (29.4% vs. 15.6% vs. 0%) was also seen in a subsequent randomized, placebo-controlled phase III trial comparing lenalidomide 10 mg/day on days 1–21 with lenalidomide 5 mg/day on days 1–28 of 28-day cycles and placebo [44]. However, there was no OS benefit with lenalidomide for the entire study population but patients who achieved RBC-TI for at least 8 weeks experienced a reduction in the relative risk of AML progression and death [44]. Although off-label, transfusion-dependent MDS patients without del(5q) also appeared to benefit from lenalidomide with or without an ESA in terms of RBC-TI although at a numerically lower rate [45,46]. Special considerations regarding lenalidomide use include the lower rate of response in patients with *TP53* mutations and its role in LR-MDS patients who are not transfusion-dependent [47,48]. The latter question is currently addressed in a randomized phase III clinical trial (NCT01243476).

The activin receptor ligand trap luspatercept interferes with signaling via the transforming growth factor (TGF)-β pathway, which has been associated with ineffective erythropoiesis in MDS [49,50,51]. Luspatercept was initially tested in the single-arm phase II PACE-MDS trial and showed rates of HI-E and RBC-TI of 63% and 38%, respectively, in LR-MDS and CMML patients treated with luspatercept with higher response rates in patients with ring sideroblasts and those with *SF3B1* and spliceosome mutations [52]. This led to the randomized, double-blind, placebo-controlled, phase III MEDALIST trial that enrolled 229 LR-MDS patients with transfusion-dependence or who were refractory or unlikely to respond to ESA and randomized participants in a 2:1 ratio to luspatercept or placebo [7]. The primary outcome of RBC-TI for ≥8 weeks was reached by 38% in the luspatercept group and 13% with placebo (*p* < 0.001), with an overall favorable safety profile [7]. Subgroup analyses of the MEDALIST trial showed that RBC-TI was achieved independent of co-mutations (including high-risk mutations), did not impact quality of life, had comparable efficacy in patients with MDS/MPN-RS-T, and appeared to yield improvements in platelet (HI-P) and neutrophil counts (HI-N) [53,54,55,56]. Based on those results, luspatercept has been approved by the United States Food and Drug Administration for ESA-refractory, transfusion-dependent patients with MDS with ring sideroblasts or MDS/MPN-RS-T. Whether luspatercept is also effective in ESA-naïve, LR-MDS patients and in those without ring sideroblasts, is currently being studied in the randomized phase III COMMANDS trial (NCT03682536) [57].

Immunosuppressive therapy primarily with cyclosporine A and anti-thymocyte globulin (ATG) can be an effective therapy for anemia in selected patients with LR-MDS. In the only randomized trial comparing ATG with or without cyclosporine A, hematologic responses were seen in 29% of patients in the combination arm vs. 9% in the ATG monotherapy arm (*p* = 0.016) [58]. Slightly higher response rates (ORR of 48.8% with 30% RBC-TI) have been reported for various immunosuppressive therapy regimens in a retrospective multicenter study with ATG + cyclosporine A being the most effective regimen as well as a systematic review and meta-analysis of 22 studies (ORR 42.5%, 33.4% RBC-TI) [59,60]. However, data on biomarkers predicting response to immunosuppressive therapy is mixed but the National Comprehensive Cancer Network (NCCN) recommends that patients ≤60 years, with ≤5% bone marrow blasts, hypocellular bone marrow, PNH clones, or STAT-3 mutant T-cell clones should be considered for immunosuppressive therapy [2,59,61].

While anemia is the most common symptom in MDS patients, neutropenia and thrombocytopenia occur in 15–20% and 50% of patients with MDS, respectively [62,63]. Supportive care with G-CSF can be considered for selected patients with neutropenia in the setting of recurrent infections. The thrombopoietin (TPO) mimetics romiplostim and eltrombopag have been evaluated in various clinical trials and have yielded platelet responses in 46–61% of patients with a reduction in bleeding events and no increase in AML transformation rate [64,65,66,67]. Recent trials have especially focused on eltrombopag alone or in combination with AZA. In a randomized phase II trial of patients with LR-MDS and thrombocytopenia (platelet count <30 × 10^9^/L) comparing eltrombopag with placebo, platelet responses by week 24 were seen in 47% vs. 3% (odds ratio 27.1 [95% CI 3.5–211.9], *p* = 0.0017), which also led to a reduction in bleeding events with eltrombopag [66]. While grade 3/4 adverse events were more common with eltrombopag (46% vs. 16%; *p* = 0.0053), the risk of AML transformation was similar (12% vs. 16%; *p* = 0.81) [66]. Similar results have been reported from another randomized phase II trial of eltrombopag vs. placebo in HR-MDS or AML patients with thrombocytopenia (platelet count <25 × 10^9^/L) that showed a reduction in clinically relevant thrombocytopenic events (defined as a composite of grade ≥3 hemorrhagic adverse events, platelet counts <10 × 10^9^/L or platelet transfusions) with eltrombopag (54% [95% CI 43–64%] vs. 69% [95% CI 57–80%]; odds ratio 0.20 [95% CI 0.05–0.87]; *p* = 0.032) [67]. Conversely, the combination of AZA and eltrombopag was inferior to AZA alone in a randomized phase III trial (NCT02158936) of HR-MDS patients with thrombocytopenia (platelet count <75 × 10^9^/L) in terms of platelet transfusion independence (16% vs. 31%) and ORR (20% vs. 35%) without any differences in hematologic improvement in any cell line but higher rates of adverse events [68]. Given the conflicting results, it is important to note that TPO mimetics have not been approved for the treatment of thrombocytopenia in MDS yet and additional clinical trials are necessary (e.g., NCT01286038, NCT01893372).

The hypomethylating agents (HMA) azacitidine (AZA) and decitabine (DEC) are only approved in the US but not in Europe for the treatment of LR-MDS and have been reported to achieve RBC-TI rates of 16–32% and cytogenetic responses in up to 61% of patients in clinical trials [69,70]. However, they are mostly reserved for the second-line setting and for younger patients with higher risk genetic features.

### 3.2. Higher-Risk MDS

While symptomatic management and supportive care are the mainstay of therapy for patients with LR-MDS, patients with higher-risk MDS (HR-MDS; i.e., IPSS-R > 3.5) have a substantial risk of progression to AML and a reduced life-expectancy warranting a more aggressive, disease-modifying approach [5,33]. A proposed treatment algorithm is presented in Figure 3.

Similar to LR-MDS patients, high-quality supportive care based on the presence of cytopenias and symptoms with ESA, blood product transfusion, iron chelation therapy, and antimicrobial prophylaxis in neutropenic patients is of paramount importance also in HR-MDS patients who are even more likely to experience symptoms of bone marrow failure [1,2,33]. However, the use of TPO mimetics and G-CSF should be carefully considered due to concern about the increase in blast counts and potentially accelerated AML transformation with growth factor use [64,71]. While more data are needed for a final assessment, more recent data suggest that TPO mimetics are not related to higher rates of AML [72,73].

Allogeneic hematopoietic cell transplant (allo-HCT) remains the only potentially curative therapeutic modality for MDS and should be considered for all eligible patients with HR-MDS and potentially even for LR-MDS with adverse genetic features such as *TP53* mutations or complex karyotypes [12,74]. Recent data from the European Society of Blood and Bone Marrow Transplantation registry have reported rates of 5-year and 10-year OS of 43% and 35%, respectively [75]. However, the non-relapse mortality at 10 years was similarly high at 34% which highlights the need for careful patient selection [75]. While advanced age has been associated with higher rates of peri-HCT mortality, the wider use of reduced-intensity conditioning regimens has increased the number of eligible patients and the safety of allo-HCT in patients older than 70 years has been shown as well [75,76]. The optimal timing of referral for allo-HCT (i.e., before or after HMA failure) and the role of pre-transplant cytoreductive therapy with intensive chemotherapy or HMA remains debatable [74]. General recommendations include consideration of allo-HCT in patients experiencing HMA failure and to use cytoreductive therapy prior to allo-HCT to achieve a bone marrow blast count of <10% as higher pre-transplant blast percentage has been shown to negatively impact outcomes [74,77]. However, it is important to note that patients with certain high-risk genetic features such as *TP53* or RAS pathway mutations remain at high risk of relapse even after transplant and that the median OS among patients with HMA failure undergoing allo-HCT in clinical trials has been only 19.5 months [78,79,80].

For the majority of HR-MDS patients, the HMAs AZA and DEC remain the mainstay of frontline therapy. AZA has been the only agent shown to have a statistically significant OS benefit in randomized clinical trials in MDS based on data from the AZA-001 trial [81]. Compared to conventional care regimens (best-supportive care, low-dose cytarabine, intensive chemotherapy), AZA led to a 9.5 month OS benefit (24.5 months vs. 15.0 months; *p* < 0.001) with ORR of 51% but only 17% achieving a CR [81]. However, this OS benefit has been more nuanced in subsequent clinical trials and real-world registry studies [82,83,84]. Response predictors to HMA have not been consistently identified but include better performance status, absence of adverse cytogenetics, and lower transfusion burden, as well as *DNMT3A* and *TET2* mutations [22,85,86]. Unlike AZA, DEC has not been shown to have an OS benefit but demonstrated a higher response rate, prolonged time to AML progression, and improvements in quality of life in randomized clinical trials [87,88]. It is important to emphasize that adherence to the approved HMA administration schedule and continuation of therapy following achievement of response is important as premature treatment discontinuation or extended treatment interruptions might lead to a loss of response that may not be regained upon resumption of therapy [89,90].

In an attempt to improve response rates to HMA, as well as to increase patient comfort by oral administration, several novel HMAs have been developed [13]. Guadecitabine is a DEC analog that is resistant to degradation by cytidine deaminase and could therefore lead to prolonged exposure and more sustained epigenetic effects [91]. In an open-label phase I/II trial of 105 patients with HR-MDS, guadecitabine had an ORR of 51% of treatment-naïve and 43% in HMA-failure patients [91]. However, the subsequent randomized phase III trial (ASTRAL-3; NCT02907359) comparing guadecitabine with physicians’ choice of low dose cytarabine, standard intensive chemotherapy (7 + 3 regimen of cytarabine and an anthracycline) or best supportive care only has reportedly been negative with regard to the primary outcome of OS although the results have not been published in a peer-reviewed journal yet and subgroup and secondary endpoint analysis might be informative [92].

ASTX727 is an oral DEC analog that combines DEC with the cytidine deaminase inhibitor cedazuridine, which inhibits DEC degradation in the gastrointestinal tract and increases its oral bioavailability. In a recent randomized, cross-over trial ASTX727 showed comparable bioavailability to DEC with an ORR of 62% and 16% CRs leading to the FDA approval of ASTX727 [93]. An oral, but not bioequivalent formulation of AZA (CC-486), has recently been approved for maintenance therapy in AML patients in CR following intensive chemotherapy who are not proceeding to allo-HCT, but data in MDS are limited [94]. In a phase II study of 31 patients (18 MDS, 4 CMML, 9 AML), ORR among MDS/CMML patients was 32% with 33% RBC-TI and a safety profile that was comparable to injectable AZA [95]. Results from another trial using either a 14-day or 21-day dosing schedule of CC-486 in patients with LR-MDS showed ORR of up to 46%, however, with a substantial burden of adverse events (grade 3/4 up to 48%) [96]. An additional study highlighted the efficacy of CC-486 in patients with baseline thrombocytopenia [97]. However, the role of CC-486 in MDS will need to be further defined by the final results of the phase III trial of CC-486 vs. placebo in transfusion-dependent LR-MDS patients (NCT01566695) that has fully accrued.

Attempts to increase response rates of HMA monotherapy in MDS have largely been unsuccessful in randomized clinical trials combining AZA with lenalidomide or histone deacetylase inhibitors such as vorinostat or entinostat [82,98]. However, several promising new combination therapies have been evaluated recently. The BCL-2 inhibitor venetoclax has been approved in combination with HMA or low-dose cytarabine for the frontline treatment of older and chemotherapy-ineligible patients with AML and is currently being studied in combination with AZA in the HMA-failure and HMA-naïve setting in MDS [99,100]. In a phase Ib study of 78 HMA-naïve HR-MDS patients, the combination of venetoclax and AZA led to an ORR of 79% with 39.7% CRs and 65% transfusion independence [101]. With a median time on the study of 16.4 months, the 24-month OS estimate was 59.6% (95% CI: 43–72.8%), which compares favorably to historic controls of AZA monotherapy including the AZA-001 trial [81,101]. However, 96% of patients experienced grade 3/4 adverse events including 49% febrile neutropenia, which highlights the added myelosuppressive effect of venetoclax [101]. In a similar trial of 44 patients with R/R-MDS, AZA + venetoclax showed an ORR of 39% with 7% CRs and 32% marrow CR (mCR; 43% of those with hematologic improvement) and a median OS of 12.3 months [102]. Interestingly, OS was independent of the IPSS-R risk category and blast count percentage with *TP53* mutations being the only marker associated with inferior OS [102]. While those results appear encouraging, it is important to await the completion of larger, randomized trials to confidently assess whether venetoclax-based combinations can be a safe and effective option in MDS. 

Additional combination therapies using an HMA backbone in combination with immune checkpoint inhibitors have been presented. Small, single-arm studies suggested additive effects for combinations of HMA with immune checkpoint inhibitors. However, those results could not be replicated in a randomized phase II trial of HMA-naïve, older MDS and AML patients. In this trial, the addition of the anti-PD-L1 inhibitor durvalumab to AZA did not improve ORR, median OS, or PFS compared to AZA monotherapy [103,104,105,106]. Several large randomized trials in the frontline, HMA-naïve setting that combine HMAs with the anti-CD47 antibody magrolimab (ENHANCE trial; NCT04313881), the anti-TIM3 antibody sabatolimab (MBG-453; STIMULUS program; e.g., NCT03946670, NCT04266301), or the anti-CD70 antibody cusatuzumab (NCT04264806) are ongoing. Finally, the neural precursor cell expressed, developmentally downregulated 8 (NEDD8)-activating enzyme inhibitor pevonedistat is currently being tested in randomized phase III trials in combination with AZA but did not show a difference in OS (21.8 vs. 19.0 months; HR 0.80; 95% CI 0.51–1.26; *p* = 0.334) [107]. However, several secondary endpoints such as EFS, progression to AML, higher rates of transfusion independence, and lower transfusion burden seemed to favor the combination arm [107]. Additionally, greater benefits in patients with high and very high-risk MDS have been reported in subgroup analyses [107].

Intensive chemotherapy with anthracycline/cytarabine-based regimens can be an effective option for patients failing HMA and as a bridge to allo-HCT [12,108]. In the absence of direct comparisons with HMAs, the rates of ORR and CR with intensive chemotherapy and HMA in the frontline setting appear comparable and patients with adverse genetic features appear to be less sensitive to intensive chemotherapy but might derive benefit from HMA [81,109,110]. CPX-351, a liposomal formulation of cytarabine and anthracycline, has been approved for newly-diagnosed therapy-related AML or AML with myelodysplasia-related changes [111]. Whether it is effective in HR-MDS patients is currently being studied in clinical trials but its role—if any—is likely limited to the HMA-failure setting although both frontline (NCT03572764, NCT04273802) and relapsed/refractory trials are ongoing (NCT04109690, NCT03957876).

## 4. Treatment Algorithm for CMML

**Figure 4:**
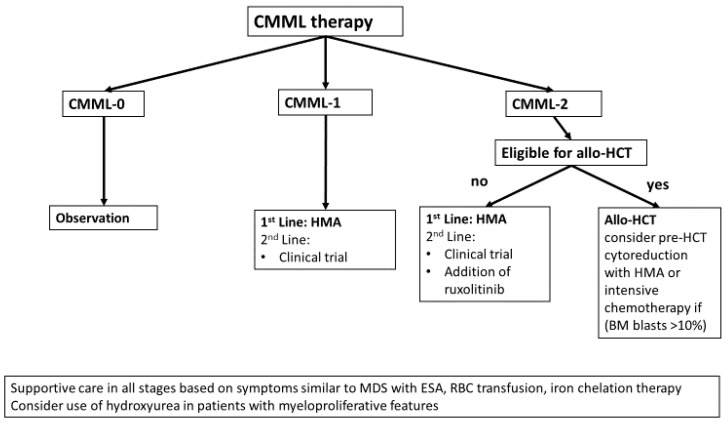
Potential treatment algorithm for CMML. Treatment of CMML should be individualized based on bone marrow and peripheral blast percentage. CMML-0 (<2% blasts in blood and <5% in bone marrow) is managed with observation. For CMML-1 (2–4% blasts in blood and 5–9% in bone marrow) and CMML-2 (5–19% blasts in blood and 10–19% in bone marrow), HMA are the only approved therapy. Especially for CMML-2, allo-HCT should be considered. The addition of ruxolitinib or clinical trial enrollment are additional options. All patients should receive supportive care similar to MDS patients based on their symptom burden with ESA, RBC transfusion, iron chelation, and growth factor support. Hydroxyurea remains a cornerstone of therapy in patients with prominent myeloproliferative disease features.

Dedicated trials in CMML patients are very rare and AZA and DEC remain the only agents approved for CMML in the US based on the inclusion of a small number of CMML patients in the pivotal AZA-001 and CALBG studies [10,81,112]. However, treatment with hydroxyurea in CMML patients with advanced myeloproliferative features remains another cornerstone of therapy. Figure 4 illustrates a potential treatment algorithm for CMML patients.

The efficacy of HMA in CMML is overall comparable with results from MDS studies. In a recent phase II study of DEC in 43 higher-risk CMML patients from Italy, the ORR was 47.6% with 16.6% CRs and a median OS of 17 months [113]. Similar but variable results have been reported from retrospective case series, clinical trial subgroup analyses, and population-based studies that reported median OS of 17–24 months and ORR of 25–71% (CR: 10–41%) although the patients included in those studies are rather heterogeneous in terms of disease risk, treatment, and demographic characteristics [114,115,116,117,118]. Identifying patients who are more likely to benefit from HMA is challenging but based on a large retrospective analysis of 949 CMML patients (412 treated with HMA), patients with higher-risk CMML by CPSS and those with myeloproliferative CMML appeared to benefit the most [115]. On a molecular level, no mutations (including *ASXL1* and *TET2*) consistently predicted response or survival in DEC-treated CMML patients [118,119,120]. 

While several new, CMML-specific therapies are in development and are being discussed in the future directions section, none of those has garnered regulatory approval yet and allo-HCT remains the only potentially curative therapy. Similar to MDS, the timing of allo-HCT referral and patient selection needs to be carefully evaluated given the potential risk of transplant-associated morbidity and mortality [74]. In the absence of prospective studies, data on the safety and efficacy of allo-HCT in CMML is derived only from retrospective studies. 5-year OS varies by baseline CPSS risk category and ranges between 44–68% and 19–40% for low/intermediate-1 and intermediate-2/high risk patients, respectively, and appears superior to non-transplant strategies in higher-risk patients with a 37% reduction in the hazard for death [121,122,123]. However, risk stratification by CPSS alone appears to have limitations [122]. Other prognostic factors predicting outcomes following allo-HCT include baseline performance status, abnormal karyotype, and graft source (inferior survival with bone marrow grafts) [121,123]. Although data are limited, pre-transplant HMA did not appear to improve outcomes in a retrospective single-center study, and strategies to optimize timing, non-transplant mortality, and relapse rates are needed [123].

## 5. Future Directions

Several novel therapies for both MDS and CMML are currently being evaluated in clinical trials (Table 1) [35]. The telomerase inhibitor imetelstat is being tested in the phase II/III IMerge trial (NCT02598661) that is enrolling ESA-refractory, transfusion-dependent LR-MDS patients. Preliminary data from the phase II part of the trial showed a 42% 8-week RBC-TI rate and a 32% 24-week RBC-TI rate but data from the randomized, placebo-controlled phase III portion are not available yet [124]. Another agent for the treatment of anemia in LR-MDS patients is roxadustat, an oral hypoxia-inducible factor (HIF)-prolyl hydroxylase inhibitor. The HIF pathway has been implicated in the regulation of hematopoiesis and roxadustat has been shown to increase hemoglobin and EPO levels as well as reductions in hepcidin in patients with chronic kidney disease in phase III trials [125]. In MDS, roxadustat is currently being studied in a phase II/III clinical trial of transfusion-dependent LR-MDS patients with serum EPO levels of <400 mIU/L (NCT03263091). Interim results of 24 enrolled patients have shown 8-week and 20-week RBC-TI of 38% and 17%, respectively, with efficacy across MDS subtypes and baseline EPO levels [126].

Thanks to the wider availability of molecular testing and advances in our understanding of the underlying disease biology, molecularly targeted therapies are also in development. Based on promising phase I/II data, APR-246, a p53-refolding agent, has been tested in a randomized phase III trial in *TP53-*mutant MDS, CMML, or AML (<30% blasts) patients in combination with AZA vs. AZA monotherapy. While single-arm studies showed ORR of 62–73% (47–50% CR) among *TP53-*mutated, HMA-naïve patients with MDS and CMML, the manufacturer has recently announced that the randomized phase III trial failed to reach its primary endpoint of OS but the publication of trial results needs to be awaited to evaluate if there are any subgroups who might benefit from APR-246 + AZA [127,128]. Similarly, the combination of the anti-CD-47 antibody magrolimab, in combination with AZA, is currently being studied in a randomized phase III trial against AZA monotherapy. While data from the phase I studies appear encouraging (ORR of 91% [30 out of 33 evaluable patients] in MDS with 42% CR rate), the reportedly negative results from the APR-246 trial should serve as a sign of caution [104]. Similar data have been reported for the combination of the anti-TIM3 antibody MBG453 with AZA in phase I trials that are currently being further evaluated in a randomized phase III trial [105,129]. Other promising targeted therapies include the IDH1/2 inhibitors ivosidenib and enasidenib which are either tested as monotherapy or in combination with AZA in clinical trials currently (NCT03383575, NCT03744390, NCT03503409). Results from small, early phase clinical trials showed efficacy even in HMA-failure patients [130]. However, it is important to note that *IDH1/2* mutations are rare in MDS and CMML and larger confirmatory trials are needed prior to routine use [10,18]. Nonetheless, the trend towards a more individualized, molecularly driven approach to patient care is likely going to continue.

Due to the rarity of the disease, dedicated trials in CMML patients have been difficult to conduct. However, several novel agents are currently being studied in early phase trials in CMML patients [10]. Tipifarnib is a farnesyltransferase inhibitor that is being studied in a phase II trial in CMML patients (NCT02807272) and was found to be well-tolerated but had only limited efficacy (1 out of 7 evaluable patients each with marrow and symptom response; other patients with stable or progressive disease) [131]. The anti-CD123 antibody tagraxofusp (SL-401) has been studied in a phase I/II trial (NCT02268253) with preliminary results from 18 HMA-refractory CMML patients and led to a spleen response in all patients (8 out of 8 patients with baseline splenomegaly) and mCRs in 2 patients [132]. Finally, the anti-GM-CSF antibody lenzilumab has been shown to be safe and moderately effective with an ORR of 33.3% by MDS/MPN IWG response criteria in a phase I trial of 15 CMML patients (NCT02546284) [133]. Notably, 3 out of 5 responses were seen in patients with *NRAS* mutations, which highlights that not only in MDS and AML but also in CMML patients, an individualized approach to treatment selection might be possible [133].

While most clinical trials in MDS use the MDS IWG 2006 response criteria, it has become increasingly clear that some of those criteria may not adequately capture patient-centered long-term outcomes [134]. For example, the RBC transfusion burden in LR-MDS patients might be fluctuating over time as evidenced by the 13% response rate seen with placebo in the MEDALIST trial [7]. As such short-term reductions in transfusion needs may not translate into long-term benefits and can lead to erroneously high response rates and trial eligibility, the IWG has proposed new response criteria with longer periods of transfusion-independence [135]. Similarly, it has been shown that mCR without hematologic improvement (HI) is prognostically similar to progressive disease in MDS patients and is inferior to HI or partial remission among HMA-treated MDS patients [136]. Especially with more myelosuppressive therapies such as venetoclax + AZA including mCR in the ORR could lead to an inflation of the ORR that does not correlate with OS. Furthermore, patient-centered outcomes such as a reduction in transfusion needs that are associated with HI are not captured by mCR. However, such revisions to clinical trials will take several years to implement but may allow for a better assessment of the benefits of a given novel therapy.

## 6. Conclusions

MDS and CMML are heterogenous disorders and management decisions should be guided by individual patient and disease characteristics. While supportive care is essential for all MDS patients and remains the mainstay of therapy for LR-MDS patients, luspatercept has recently been approved for transfusion-dependent anemic LR-MDS patients and several additional agents are undergoing advanced stages of clinical testing. Allo-HCT remains the only curative therapy for both MDS and CMML but despite the more frequent use of reduced-intensity conditioning regimens and alternative grafts, as well as advances in supportive care, the majority of patients are not eligible for allo-HCT and are treated with HMA. AZA remains the only agent that has shown an OS benefit in MDS and HMA monotherapy remains the standard of care for frontline management of HR-MDS. Molecularly driven agents such as IDH1/2 inhibitors, venetoclax, magrolimab, and APR-246 for MDS, as well as tagraxofusp, tipifarnib, and lenzilumab for CMML are being evaluated in various stages of clinical trials but more data are needed prior to their use in routine clinical practice. 

## Figures and Tables

**Table 1:** Selected active phase II/III trials of novel agents in MDS and CMML.

Drug	Phase	NCT	Patient Characteristics	Intervention
**Hypomethylating Agents**				
Decitabine	III	NCT02214407 (GFM-DAC-CMML)	CMML	DEC + hydroxyurea vs. hydroxyurea alone
Azacitidine	II	NCT01522976	HR-MDS or CMML	AZA +/− lenalidomide or vorinostat
	I/II	NCT00392353	HR-MDS, CMML or AML	AZA + vorinostat
Guadecitabine	I/II	NCT02935361	R/R MDS or CMML	Guadecitabine + atezolizumab
	III	NCT02907359 (ASTRAL-3 trial)	HMA-refractory MDS or CMML	Guadecitabine vs. treatment choice (low-dose cytarabine, BSC, 7 + 3)
CC-486	II	NCT02281084	HMA-refractory MDS	CC-486 + durvalumab vs. CC-486 alone
	III	NCT01566695	Transfusion-dependent LR-MDS	CC-486 vs. placebo
	III	NCT04173533 (AMADEUS trial)	AML and MDS post-HSCT maintenance therapy	CC-486 vs. placebo
ASTX030	II/III	NCT04256317	MDS, CMML, MDS/MPN, or AML who are candidates for AZA monotherapy	Phase 2: randomized open-label crossover study oral ASTX030 vs. subcutaneous AZA Phase 3: randomized open-label crossover study of final oral ASTX030 tablet vs. subcutaneous AZA
ASTX727	III	NCT03306264	HR-MDS, CMLL, or AML	ASTX727 vs. IV DEC
	I/II	NCT04061421	MDS/MPN overlap except JMML	ASTX727 + INCB053914, itacitinib, or INCB059872
	I/II	NCT03502668	RBC-TD LR-MDS	Low-dose vs. standard-dose ASTX727
	II	NCT04655755	Newly diagnosed HR-MDS or CMML	ASTX727 + venetoclax
	II	NCT04093570	Any prior enrollment in ASTX727 trials	ASTX727
**Molecularly Targeted Agents**				
APR-246 (p53-refolding agent)	III	NCT03745716	*TP53-*mutant MDS	APR-246 + AZA vs. AZA alone
	II	NCT03931291	*TP53-*mutant MDS or AML following allo-HCT	APR-246
	I/II	NCT03072043	*TP53-*mutant MDS, CMML or AML	APR-246 + AZA
	I/II	NCT03588078	*TP53-*mutant MDS, CMML or AML	APR-246 + AZA
Quizartinib (FLT3 inhibitor)	I/II	NCT01892371	R/R AML, MDS, CMML	Quizartinib + AZA
	I/II	NCT04493138	Untreated or HMA-refractory MDS, MDS/MPN with *FLT3* or *CBL* mutations	Quizartinib + AZA
	II	NCT04047641	Untreated or R/R AML or HR-MDS with *FLT3* mutations	Cladribine + idarubicin + cytarabine + quizartinib
Gilteritinib (FLT3 inhibitor)	III	NCT04027309 (HOVON 156 AML)	Untreated AML or HR-MDS with *FLT3* mutations	Gilteritinib + induction chemotherapy vs. midostaurin + induction chemotherapy
Ivosidenib (IDH1 inhibitor)	II	NCT03503409	*IDH1* Mutated, HMA-refractory MDS	Ivosidenib
	II	NCT03471260	*IDH1* Mutated MDS, MPN, AML	Ivosidenib + venetoclax +/− AZA
	III	NCT03839771 (HOVON150AML)	*IDH1* Mutated newly diagnosed and R/R-AML and HR-MDS	Ivosidenib or placebo in combination with induction and consolidation therapy
Enasidenib (IDH2 inhibitor)	II	NCT03744390	*IDH2* Mutated MDS	Enasidenib
	II	NCT03383575	*IDH2* Mutated, HMA-naïve and HMA-refractory MDS	Enasidenib + AZA or enasidenib alone in HMA-refractory patients
	III	NCT03839771 (HOVON150AML)	*IDH2* Mutated newly diagnosed and R/R-AML and HR-MDS	Ivosidenib or placebo in combination with induction and consolidation therapy
	II	NCT01915498	*IDH2* Mutated R/R-AML and HR-MDS	Enasidenib
FT-2102 (IDH1 inhibitor)	II	NCT02719574	*IDH1* Mutated R/R-AML and HR-MDS	FT-2102 alone or in combination with AZA or cytarabine
**Immune Checkpoint Inhibitors/Cellular Immunotherapy/Monoclonal Antibodies**				
MBG453 (anti-TIM3)	II	NCT03946670	HMA-naïve, HR-MDS	MBG453 + HMA vs. placebo + HMA
	III	NCT04266301 (STIMULUS-MDS2)	HMA-naïve, HR-MDS	MBG453 + AZA vs. placebo + AZA
Nivolumab (anti-PD1)	I/II	NCT02530463	Untreated or HMA-refractory MDS	Nivolumab +/− ipilimumab +/− AZA
	II/III	NCT03092674	Untreated AML or HR-MDS	AZA +/− nivolumab or midostaurin vs. DEC/cytarabine
Durvalumab (anti-PD-L1)	II	NCT02775903	Untreated HR-MDS or AML ≥65 years old and not eligible for allo-HCT	Durvalumab + AZA vs. AZA alone
Pembrolizumab (anti- PD1)	II	NCT03094637	Untreated or HMA-refractory MDS	Pembrolizumab + AZA
Ipilimumab (anti-CTLA4)	Ib/II	NCT02890329	R/R-AML and MDS	Ipilimumab + DEC
Magrolimab (anti-CD47)	III	NCT04313881 (ENHANCE)	Untreated HR-MDS	Magrolimab + AZA vs. placebo + AZA
ALX148 (anti-CD47)	I/II	NCT04417517 (ASPEN-02)	HR-MDS	ALX148 + AZA
TJ011133 (anti-CD47)	II	NCT04202003	R/R-AML or MDS	TJ011133
Cusatuzumab (anti-CD27/70)	II	NCT04264806	HR-MDS and CMML	Cusatuzumab + AZA vs. AZA alone
	II	NCT03030612	Newly-diagnosed AML or HR-MDS ineligible for chemotherapy	Cusatuzumab + AZA
BLEX 404 (immune stimulant)	II	NCT02944955	Intermediate-1, Intermediate-2 or High-Risk MDS and CMML	BLEX404 + AZA
Talacotuzumab (JNJ-56022473; anti-CD123) or Daratumumab (anti-CD38)	II	NCT03011034	RBC-TD LR-MDS	Talacotuzumab (JNJ-56022473) or Daratumumab
Daratumumab (anti-CD38)	II	NCT03067571	R/R-AML or HR-MDS	Daratumumab
ADCT-301 (anti-CD25 antibody drug conjugate)	II	NCT04639024	R/R-AML, MDS, or MDS/MPN	ADCT-301
ASP7517 (tumor vaccine)	II	NCT04079296	R/R-AML or MDS	ASP7517
Canakinumab (anti-IL-1β)	II	NCT04239157	ESA or HMA-refractory LR-MDS or CMML	Canakinumab
SAR440234 (CD3-CD123 T-cell engaging bispecific monoclonal antibody)	II	NCT03594955	R/R AML, ALL or HR-MDS	SAR440234
**Conventional Cytotoxic Chemotherapy **				
CPX-351 (liposomal cytarabine + daunorubicin)	I/II	NCT04109690	HMA-refractory MDS	CPX-351
	II	NCT03957876	HMA-refractory MDS	CPX-351
	I/II	NCT04273802	Untreated or HMA-refractory MDS	CPX-351
	I/II	NCT04128748	Frontline and R/R AML and MDS	CPX-351 + quizartinib
	II	NCT04668885	R/R AML and MDS	CPX-351
	II	NCT04493164	Frontline and R/R AML and MDS with *IDH1* mutation	CPX-351 + ivosidenib
	II	NCT03672539	R/R AML or HR-MDS	CPX-351 + gemtuzumab ozogamicin
BST-236 (cytarabine prodrug)	II	NCT04749355	R/R-AML or HMA-failure, HR-MDS; MDS/MPN overlap excluded	BST-236
**Small Molecule Inhibitors and Miscellaneous Agents **				
Pevonedistat (NEDD8 inhibitor)	II	NCT03238248	HMA-refractory MDS or MDS/MPN	Pevonedistat + AZA
	III	NCT03268954 (PANTHER)	Newly-diagnosed HR-MDS, CMML or AML <30% blasts	Pevonedistat + AZA vs. AZA alone
	II	NCT03238248	HMA-refractory MDS or MDS/MPN	Pevonedistat + AZA
Venetoclax (BCL2 inhibitor)	II	NCT04146038	R/R-AML or MDS	Salsalate + DEC/AZA + venetoclax
	I/II	NCT03661307	Frontline and R/R, AML and MDS	DEC + venetoclax + quizartinib
	I/II	NCT04140487	R/R, FLT3-mutated AML and MDS	Venetoclax + AZA + gilteritinib
	II	NCT04487106	R/R, RAS pathway-mutated AML and MDS	Venetoclax + AZA + trametinib
	I/II	NCT03218683	R/R AML or MDS	AZD5991 +/− venetoclax
	II	NCT03404193	R/R AML and MDS	Venetoclax + DEC
	I/II	NCT04550442	HMA-refractory MDS and CMML	Venetoclax + AZA
	I/II	NCT04160052	Frontline and R/R HR-MDS	Venetoclax + AZA
	II	NCT02115295	Frontline or R/R AML or HR-MDS	Cladribine + idarubicin + cytarabine + venetoclax
	III	NCT04401748 (VERONA trial)	Newly diagnosed HR-MDS	Venetoclax + AZA vs. AZA + placebo
	III	NCT04628026	Newly diagnosed AML or HR-MDS	Venetoclax + induction chemotherapy vs. placebo + induction chemotherapy
BGB-11417 (BCL2 inhibitor)	II	NCT04771130	Newly-diagnosed AML, MDS, or MDS/MPN overlap	BGB-11417 + AZA
Rigosertib (PLK1 inhibitor)	III	NCT02562443 (INSPIRE trial)	HMA-refractory HR-MDS	Rigosertib vs. treatment choice
	II	NCT01904682	RBC-TD LR-MDS	rigosertib
	II	NCT01926587	HR-MDS, CMML, or AML <30% blasts	Rigosertib + AZA
Roxadustat (HIF1α inhibitor)	III	NCT03263091	Very Low, Low or Intermediate IPSS-R With <5% Blasts) MDS with low-transfusion burden	Roxadustat vs. placebo
Imetelstat (telomerase inhibitor)	II/III	NCT02598661 (IMerge trial)	LR-MDS, ESA-refractory	Imetelstat vs. placebo
Recombinant TPO	II/III	NCT04324060	LR-MDS with thrombocytopenia	Danazol +/− recombinant human TPO
Eltrombopag (TPO mimetic)	II	NCT00961064	LR-MDS with thrombocytopenia	Eltrombopag
	II	NCT02912208	LR-MDS with thrombocytopenia	Eltrombopag vs. placebo
	II	NCT01286038	HMA-refractory MDS, MDS/MPN overlap, AML <30% blasts with thrombocytopenia	Eltrombopag
	II	NCT01772420	LR-MDS with symptomatic anemia	Eltrombopag + lenalidomide
Glasdegib (hedgehog pathway inhibitor)	II	NCT01842646	MDS, CMML, or AML with <30% bone marrow blasts with HMA failure	Glasdegib
	II	NCT02367456 (BRIGHT 1012)	Untreated MDS, CMML, or AML ineligible for intensive chemotherapy	Glasdegib + AZA
Luspatercept (TGFβ pathway inhibitor)	III	NCT03682536	RBC-TD, ESA-naïve LR-MDS	Luspatercept vs. Epoetin alfa
	III	NCT02631070 (MEDALIST)	RBC-TD, ESA-resistant LR-MDS with ≥15% ring sideroblast or ≥5% SF3B1 mutation	Luspatercept vs. placebo
	I/II	NCT04539236	RBC-TD, ESA-resistant LR-MDS	Luspatercept + lenalidomide
	IIIb	NCT04064060	MDS, myelofibrosis, beta-thalassemia previously enrolled in luspatercept clinical trials	Luspatercept
KER-050 (TGFβ pathway inhibitor)	II	NCT04419649	RBC-TD LR-MDS	KER-050
SY-1425 (selective retinoic acid receptor α agonist)	II	NCT02807558	R/R-AML or HR-MDS; frontline AML ineligible for intensive chemotherapy	SY-1425 (tamibarotene) + AZA + daratumumab
Alvocidib (CDK9 inhibitor)	Ib/II	NCT03593915	Untreated HR-MDS	Alvocidib + DEC or AZA
Selinexor (selective inhibitor of nuclear export)	II	NCT02228525	HMA-refractory MDS	Selinexor
ATG 016 (selective inhibitor of nuclear export)	II	NCT04691141	HMA-refractory HR-MDS	ATG 016
	I/II	NCT02649790	HMA-refractory, HR-MDS	KPT-8602
Bemcentinib (AXL kinase inhibitor)	II	NCT03824080	HMA-refractory MDS and AML	Bemcentinib
ONO-7475 (AXL inhibitor)	II	NCT03176277	R/R AML or MDS	ONO-7475 +/− venetoclax
LB-100 (protein phosphatase 2A inhibitor)	II	NCT03886662	HMA-refractory LR-MDS	LB-100
TEW-7197 (Vactosertib; ALK5 inhibitor)	II	NCT03074006	LR-MDS	TEW-7197
INCB000928 (ALK2 inhibitor)	II	NCT04582539	ESA-refractory MDS	INCB000928
TP-0184 (ALK2 or ACRV1 kinase inhibitor)	II	NCT04623996	ESA-refractory LR-MDS	TP-0184
Omacetaxine (protein translation inhibitor)	II	NCT03564873	Newly diagnosed, HR-MDS or CMML-2	Omacetaxine + AZA
CG200745 PPA (HDAC inhibitor)	II	NCT02737462	HMA-refractory MDS	CG200745 PPA
CPI-613 (PDH/α-KGDH inhibitor)	II	NCT03929211	HMA-refractory HR-MDS	CPI-613 + hydroxychloroquine
Ascorbic acid	II	NCT03397173	Newly diagnosed AML, MDS, or MDS/MPN overlap with *TET2* mutations	Ascorbic acid + AZA
CFI-400945 (PLK4 inhibitor)	II	NCT04730258	R/R or untreated AML, MDS, or CMML	CFI-40095 +/− AZA or DEC
ONC201 (dopamine D2 receptor antagonist)	II	NCT02392572	R/R-AML or HR-MDS	ONC201 + LDAC
Olaparib (PARP inhibitor)	II	NCT03953898	R/R-AML or HR-MDS with *IDH* mutations	Olaparib
Veliparib (PARP inhibitor)	II	NCT03289910	Newly-diagnosed or R/R-AML, CMML or MPN	Carboplatin + Topotecan +/− veliparib
Sirolimus (mTOR inhibitor)	II	NCT01869114	R/R-AML or HR-MDS	Sirolimus + AZA
IGF-MTX (methotrexate conjugate)	I/II	NCT03175978	R/R-AML or HR-MDS/CMML	IGF-methotrexate conjugate
OTS167 (MELK inhibitor)	I/II	NCT02795520	R/R AML, MDS, ALL, CML, MPN	OTS167
Ruxolitinib (JAK inhibitor)	II	NCT01787487	MDS/MPN overlap	Ruxolitinib + AZA
Seclidemstat (LSD1 inhibitor)	II	NCT04734990	HMA-refractory, HR-MDS or CMML	Seclidemstat + AZA
CB-839 (glutaminase inhibitor)	II	NCT03047993	HR-MDS	CB-839 + AZA
Tipifarnib (farnesyl transferase inhibitor)	II	NCT02807272	CMML, MDS/MPN overlap or AML	Tipifarnib
EP0042	II	NCT04581512	R/R-AML, MDS, or CMML	EP0042
